# Assessing socioeconomic inequalities in cognitive impairment among older adults: a study based on a cross-sectional survey in India

**DOI:** 10.1186/s12877-022-03076-6

**Published:** 2022-05-04

**Authors:** T. Muhammad, Shobhit Srivastava, T. V. Sekher

**Affiliations:** 1 Department of Family and Generations, International Institute for Population Sciences, Mumbai, Maharashtra 400088 India; 2 Department of Survey Research & Data Analytics, International Institute for Population Sciences, Mumbai, Maharashtra 400088 India

**Keywords:** Socioeconomic status, Cognitive impairment, Older adults, Decomposition

## Abstract

**Background:**

The rapidly aging population is a major concern for countries, especially where cognitive health in older age is poor. The study examined the socioeconomic and health-related factors associated with cognitive impairment among older adults and the contribution of those factors to the concentration of low cognitive functioning among older adults from economically poor households.

**Methods:**

Data this study were derived from the “Building Knowledge Base on Population Ageing in India” (BKPAI) survey, which was carried out in seven major states of India. The effective sample size for the analysis was 9176 older adults aged 60 years and above. Results from descriptive and bivariate analysis were reported in the initial stage. Multivariable logistic regression analysis was conducted to explore the associations. Additionally, the concentration index and concentration curve were used to measure socioeconomic inequality in cognitive impairment among older adults. Wagstaff decomposition was employed to explore the key contributors in the concentration index.

**Results:**

Nearly 60% of older adults suffered from cognitive impairment in the study. The likelihood of cognitive impairment were higher among older adults with a low level of self-perceived income sufficiency [coefficient: 0.29; confidence interval (CI): 0.07- 0.52] compared to older adults with higher levels of perceived income status. Older adults with more than 10 years of schooling were less likely to be cognitively impaired [coefficient: -1.27; CI: − 1.50- -1.04] in comparison to those with no education. Cognitive impairment was concentrated among older adults from households with the lowest wealth quintile (concentration index (CCI): − 0.10: *p* < 0.05). Educational status explained 44.6% of socioeconomic inequality, followed by 31.8% by wealth status and 11.5% by psychological health. Apart from these factors, difficulty in instrumental activities of daily living (3.7%), caste (3.7%), and perceived income sufficiency to fulfil basic needs (3.0%) explained socioeconomic inequality in cognitive impairment among older adults.

**Conclusions:**

Findings suggest that older adults with lower perceived income, lower levels of education, poor physical and mental health, and poor physical and social resources were more likely to be cognitively impaired. Education, wealth and psychological health are major contributors in socioeconomic inequality in late-life cognitive impairment, which may be target areas in future policy formulation to reduce the inequality in cognitive impairment in older Indian adults.

## Background

Due to aging of the brain and declining physical health, late-life mental disorders are expected to increase, with around 15% of older persons aged 60 years and above suffering from a mental or neurological disorder worldwide [1]. Cognitive function is defined as a set of thinking abilities that can be measured through performance-based tasks such as memory, executive function and processing speed [2]. Age-related diseases result in less resource for mental tasks and reduce cognitive resources in older individuals which may impact their daily functional abilities [3]. Given the greater socioeconomic distributional disparities in developing countries like India, it is important to understand what factors contribute to the differing levels of cognitive health among people from lower and higher socioeconomic groups.

Notably, older adults among the general population tend to experience a higher incidence of poverty and deprivation of basic services [4], whereas, the ability to deal with health problems in older age is associated with access to resources, and such access is hindered by poor socioeconomic circumstances [5], therefore, leading to increased susceptibility to the deleterious effects of aging and cognitive deficits. There is a wealth of literature showing independent association between socioeconomic status and cognitive function in later life [6–9]. However, it is documented that an adverse socioeconomic status with accumulating disadvantages reflects an increased risk of cognitive impairment [10–12]. Thus, it is recommended in health disparities research to add cumulative and aggregate measures of socioeconomic status such as education, wealth, social class, and asset ownership which may perform better than measures of current position [13]. Similarly, recent evidence suggests that self-perceived (subjective) income sufficiency is a useful indicator of individuals’ socioeconomic resources as a determinant of health [14, 15].

Education on the other hand, one of the important indicators of socioeconomic status, has been most extensively studied in the cognition research [9, 16–18]. A study that examined the influence of education on cognitive performance controlling for household economic variables, concluded that although the education-cognition relationship partially reflected an SES gradient, the association was more likely due to the process and consequences of education itself [19]. But, studies that examined the association of other socioeconomic indicators such as income, household wealth, and occupation with late-life cognition, showed inconsistency in their findings [20–23]. Throughout the literature, in India, being a rural resident, belonging to households with poor economic situations, experiencing violence, and other socio-cultural factors that make older individuals less important in their households were also found to have a negative impact on their cognitive health outcomes [12, 24–26]. Thus, with the current demographic structure in India that is evolving rapidly, increased inequality in major socioeconomic indicators across different sub-populations may be associated with inequality in older adults’ cognitive functioning.

In this regard, understanding the contribution of specific factors to the late-life cognitive inequality in a country with higher rates of illiteracy may help policymakers develop strategies targeting the sub-populations at greater risk. Therefore, in this study, we examine the socioeconomic and health-related factors associated with cognitive impairment among older adults and the contribution of those factors to the concentration of low cognitive functioning among older adults from poor households by employing a decomposition technique. Also, subjective income status as a potential covariate of the cognitive impairment among older adults that has rarely been analysed in an Indian context is included in the present study. We hypothesize that there is a significant wealth-based inequality in late-life cognitive functioning in India. Also, we hypothesize that low levels of perceived income status, poor wealth, low education and lack of asset ownership are positively associated with cognitive impairment among older Indian adults.

## Methods

### Data

Data for this study were derived from the ‘Building Knowledge Base on Population Ageing in India’ (BKPAI) survey which was carried out in India. The survey was carried out in seven major states of India (Himachal Pradesh, Punjab, West Bengal, Odisha, Maharashtra, Kerala, and Tamil Nadu), which covered a total of 9852 older adults from 8329 elderly households in rural and urban areas. These states have a higher percentage of the 60+ population compared to the national average, and these states represent all regions of the country in terms of geographical location. The individual dataset was used, which covers the socio-demographic profile, work history and benefit, income, and assets, living arrangement, social activities, the health status of the elderly & social security-related questions [27]. The BKPAI sample design entails a two-stage probability sampling, where, first, villages were classified into different strata based on population size, and the number of Primary Sampling Units (PSUs) to be selected was determined in proportion to the population size of each stratum. Using the probability proportional to population size (PPS) technique, the PSUs have been chosen, and within each selected PSU, elderly households were selected through systematic sampling. A similar procedure was applied in drawing samples from urban areas [27]. The final sample size for the analysis after removing missing cases and outliers was 9176 older adults aged 60 years and above.

### Variable description

#### Outcome variable

The outcome variable was binary and was assessed through verbal recall strategy which was used to measure cognitive functioning/impairment in previous studies [28, 29]. A scale of 0 to 10 was created to assess cognitive impairment. Lower cognitive impairment is associated with higher scores and vice versa. Bus, House, Chair, Banana, Sun, Bird, Cat, Saree, Rice, and Monkey were the nouns used to measure cognitive abilities. While dichotomizing, five or more words were recoded as 0 “low,” indicating lower cognitive impairment, and four or fewer words as 1 “high,” indicating higher cognitive impairment.

#### Equity stratifier

The household wealth index was the equity stratifier in the current study. The wealth index is created based on the BKPAI survey with the following 30 assets and housing characteristics: household electrification; drinking water source; type of toilet facility; type of house; cooking fuel; house ownership; ownership of a bank or post-office account; and ownership of a mattress, a pressure cooker, a chair, a cot/bed, a table, an electric fan, a radio/transistor, a black and white television, a colour television, a sewing machine, a mobile telephone, any landline phone, a computer, internet facility; a refrigerator, a watch or clock, a bicycle, a motorcycle or scooter, an animal-drawn cart, a car, a water pump, a thresher, and a tractor. The range of index was from poorest to richest, i.e. ranging from lowest to highest.

#### Explanatory variables

Due to a higher proportion of missing data in the objective income status, self-perceived income sufficiency was used as an indicator of individuals’ income status. It was recoded as (no income, has income and fully sufficient, has income and partially sufficient, and has income and not sufficient), work status (in last 1 year) was recoded as (never worked, currently working, and retired), educational status was recoded as (not educated, below 5 years, 6-10 years and 11+ years), marital status was recoded as (not in a union and currently in a union), asset ownership was asked regarding homeownership, land ownership, jewellery ownership, and other monetary savings and was recoded as (‘no’ and ‘yes’), age was recoded as (60-69 years, 70-79 years and 80+ years), gender was available as men and women. Co-residing with children was recoded as (‘no’ and ‘yes’).

Several health-related variables were selected based on the abovementioned literature. Self-rated health had a scale of 1 to 5 (poor to excellent) and was categorized as 0 “good” (representing good, very good, and excellent) and 1 “poor” (representing poor or fair). Ability to do activities of daily living (feeding, bathing, dressing, toileting, mobility (i.e., getting in and out of a bed or chair) and continence (controlling bladder and bowel movement)) was having a scale of 0 to 6, where, higher the score, higher the independence. A score of 6 was recoded as 0 “high” which represents full independence, and five and less was recoded as 1 “low” which represents not being fully independent to do activities of daily living (Cronbach Alpha: 0.93) [30]. The ability to do instrumental activities of daily living (IADL) had a scale of 0 to 8, representing the higher the score, higher the independence. A score of 6+ was recoded as 0 “high” representing high IADL, and a score of 5 and less was recoded as 1 “low” representing low IADL. The 12-item version of the General Health Questionnaire (GHQ-12) was used to measure the psychological health. Psychological health had a scale of 0 to 12 based on experiencing stressful symptoms and was recoded as 0 “high” (representing 6+ scores) and 1 “low” (representing score five and less) (Cronbach’s alpha: 0.90) [31, 32]. The 9-item subjective well-being questionnaire was used to measure low subjective well-being. Subjective wellbeing was having a scale of 0 to 9 and was categorized as 0 “high” experiencing better experience (representing 6+ scores) and 1 “low” experiencing negative experience (representing score 5 and less) [33]. Twelve questions on psychological health and nine questions on subjective well-being were asked to assess the outcome. All the questions were asked on Likert scales and were recoded and used accordingly as per literature (Cronbach alpha: 0.93) [34].

Religion of the respondent was recoded as Hindu, Muslim, Sikh, and others, caste was available as Scheduled Castes, Scheduled Tribes, Other Backward Classes, and others, and place of residence was either rural or urban. States were available as Himachal Pradesh, Punjab, West Bengal, Orissa, Maharashtra, Kerala, and Tamil Nadu.

#### Statistical analysis

Descriptive statistics were reported along with the results from bivariate analysis which was conducted to find the plausible associations between exposure and potential risk factors and cognitive impairment, using the chi-square test. Multivariable logistic regression [35] was used to explore the relationships between the explanatory variables and cognitive impairment. The estimates were adjusted for all the covariates considered in the study including age and education. The software used was STATA 14 [36]. The significance level was set at 5% (*p* < 0.05). The variance inflation factor (VIF) was used to check the presence of multicollinearity in the explanatory variables, which showed no evidence of multicollinearity [37].

#### Concentration index (CCI)

On the y-axis, the cumulative proportion of outcome variables (cognitive impairment) is plotted against the increasing percentage of the population ranked by the socioeconomic indicator (wealth index) on the x-axis to generate the concentration curve [38]. Such curves show whether or not socioeconomic inequality in the outcome variable (on the x-axis) prevails, with the index value being negative if the curve is above the line of equality (45-degree line), indicating that the outcome variable is disproportionally concentrated among the poor, and vice versa [39, 40]. The concentration index (CCI) and the concentration curve (CC) were used to quantify wealth-related inequality for cognitive impairment, using the wealth score as the socioeconomic indicator and the binary outcome as cognitive impairment. The concentration index is the area between the concentration curve and the line of equality multiplied by two [39, 40]. The concentration index compares the distribution of one variable (say, cognitive impairment) to another variable’s distribution (wealth index). The index runs from − 1 to + 1, with 0 (zero) indicating no socioeconomic disparities. The index’s positive score, on the other hand, indicates pro-rich inequality and vice versa [39, 40]. Furthermore, the higher the value on either scale the greater the degree of socioeconomic inequality. The concentration index was decomposed using Wagstaff decomposition methodology [39, 40]. The breakdown of the concentration index by Wagstaff illustrates that the wealth-related inequalities may be dissected into the contributions of each element [41]. For any linear regression model on a health outcome (y) (say cognitive impairment), such as.

1 $$y=\alpha +{\sum}_k{\beta}_k{x}_k+\varepsilon$$

The concentration index for y, C, can be written as follows,

2 $$C={\sum}_k\left({\beta}_k{\overline{x}}_k/\mu \right){C}_k+G{C}_{\varepsilon }/\mu$$

Where *μ* is the mean of y, ${\overline{x}}_k$ is the mean of *x*_*k*_, *C*_*k*_ is the concentration index for *x*_*k*_ (defined analogously to C), and *GC*_*ε*_ is the generalized concentration index for the error term (*ε*). Eq. [2] shows that C is equal to a weighted sum of the concentration indices of the k regressor, where the weight for *x*_*k*_ is the elasticity of y with respect to *x*_*k*_ $\left({\eta}_k={\beta}_k\frac{{\overline{x}}_k}{\mu}\right)$. The last term captures the socioeconomic inequality in health that is not explained by systematic variation in the regressor by wealth, which should approach zero for a well-specified model [39, 40]. The elasticity of each contribution is multiplied by the degree of economic disparity. Furthermore, the estimates are generated by dividing each absolute contribution by the overall absolute contribution multiplied by 100 to give the percentage contribution [39, 40].

## Results

Table 1 presents the socioeconomic and demographic profile of older adults. While 33.5% of the older adults perceived that the income was not partially or completely sufficient, about 43% of them reported that they had no income. Nearly 67% of older adults did not work in the last year. Almost half of the older adults had no education, and nearly 40% of older adults were not in a marital union. About 18% of older adults had no asset ownership. One-tenth of older adults were from the oldest old age group (80+ years). More than 50% of participants were women. About 29% of older adults were not co-residing with their children. More than half of the older adults reported that they had poor self-rated health; about 57% reported that they had low IADL and about 7% had low ADL. Nearly 27% and 24% had low subjective well-being and low psychological health. About 24% of older adults belonged to the poorest wealth status, and 15% belonged to the richest wealth status. Nearly 80% of the population belonged to the Hindu religion, and 21% belonged to the Scheduled Caste category. About 26% of the study population resided urban areas at the time of the survey.

**Table 1:** Socioeconomic and demographic profile of older adults

Variables	Sample	Percentage
**Cognitive impairment**		
No	3670	40.0
Yes	5506	60.0
**Self-perceived income sufficiency**		
Has income and fully sufficient	2156	23.5
Has income and partially sufficient	2410	26.3
Has income and not sufficient	661	7.2
No income	3949	43.0
**Work Status (last one year)**		
Never worked	6174	67.3
Currently working	2208	24.1
Retired	794	8.7
**Educational status**		
Not educated	4654	50.7
Below 5 years	1890	20.6
6 to 10 years	2072	22.6
11+ years	559	6.1
**Marital status**		
Not in union	3632	39.6
Currently in union	5544	60.4
**Asset ownership**		
No	1610	17.6
Yes	7566	82.5
**Age group (in years)**		
60-69	5667	61.8
70-79	2525	27.5
80+	984	10.7
**Gender**		
Men	4339	47.3
Women	4837	52.7
**Co-residing with children**		
No	2701	29.4
Yes	6475	70.6
**Self-rated health**		
Good	4096	44.6
Poor	5080	55.4
**IADL**		
High	3995	43.5
Low	5181	56.5
**ADL**		
High	8498	92.6
Low	678	7.4
**Subjective well-being**		
High	6720	73.2
Low	2456	26.8
**Psychological health**		
High	7024	76.6
Low	2152	23.5
**Wealth status**		
Poorest	2170	23.7
Poorer	2024	22.1
Middle	1903	20.7
Richer	1708	18.6
Richest	1370	14.9
**Religion**		
Hindu	7299	79.6
Muslims	644	7.0
Sikh	847	9.2
Others	386	4.2
**Caste**		
Scheduled Caste	1897	20.7
Scheduled Tribe	515	5.6
Other Backward Class	3353	36.5
Others	3411	37.2
**Place of residence**		
Rural	6783	73.9
Urban	2393	26.1
**State**		
Himachal Pradesh	1456	15.9
Punjab	1240	13.5
West Bengal	1127	12.3
Orissa	1453	15.8
Maharashtra	1230	13.4
Kerala	1340	14.6
Tamil Nadu	1330	14.5
**Total**	9176	100.0

*IADL* Instrumental activities of daily living, *ADL* Activities of daily living

**Fig. 1:**
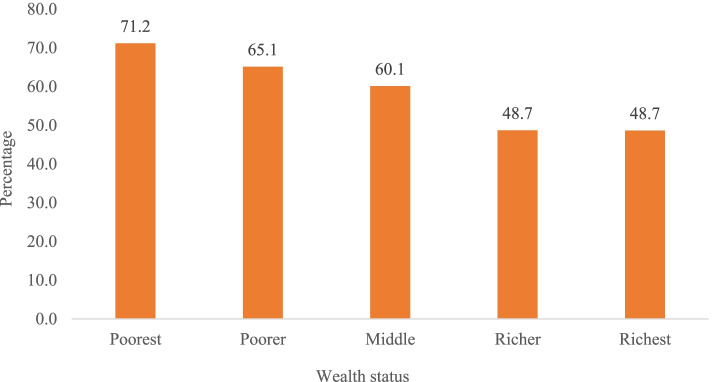
Percentage of older adults with cognitive impairment by household wealth quintile

Figure 1 presents the percentage of older adults with cognitive impairment according to socioeconomic status. It was found that cognitive impairment was highest among older adults from households with the poorest wealth quintile (71.2%) and lowest among those from households with the richest wealth quintile (48.7%).

Table 2 presents the percentage of older adults with cognitive impairment. The highest percentage of older adults who had income and was not sufficient (71.1%) were cognitively impaired. A higher percentage of older adults who never worked were cognitively impaired (66.0%). The prevalence of cognitive impairment was high among older adults who had no education (70.6%). The prevalence of cognitive impairment was high among older adults with no asset ownership (71.6%). The higher percentage of older adults with poor self-rated health (69%), low IADL (68%), low ADL (84.7%), low subjective well-being (74.6%) and low psychological health (76.5%) were cognitively impaired. The prevalence of cognitive impairment was highest in West Bengal (81.9%) followed by Orissa (69.3%) and Kerala (66.3%).

**Table 2:** Percentage of older adults with cognitive impairment

Variables	Percentage (Cognitive impairment)	***p***-value
**Self-perceived income sufficiency**		< 0.001
Has income and fully sufficient	43.5	
Has income and partially sufficient	65.6	
Has income and not sufficient	71.1	
No income	63.8	
**Work Status (last one year)**		< 0.001
Never worked	66.0	
Currently working	53.0	
Retired	32.8	
**Educational status**		< 0.001
No education	70.6	
Below 5 years	63.6	
6 to 10 years	40.8	
11+ years	31.0	
**Marital status**		< 0.001
Not in union	68.9	
Currently in union	54.2	
**Asset ownership**		< 0.001
No	71.6	
Yes	57.5	
**Age group (in years)**		< 0.001
60-69	53.1	
70-79	68.2	
80+	78.5	
**Gender**		< 0.001
Men	53.0	
Women	66.3	
**Co-residing with children**		0.006
No	57.8	
Yes	60.9	
**Self-rated health**		< 0.001
Good	48.9	
Poor	69.0	
**IADL**		< 0.001
High	49.6	
Low	68.0	
**ADL**		< 0.001
High	58.0	
Low	84.7	
**Subjective well-being**		< 0.001
High	54.7	
Low	74.6	
**Psychological health**		< 0.001
High	55.0	
Low	76.5	
**Wealth status**		< 0.001
Poorest	71.2	
Poorer	65.1	
Middle	60.1	
Richer	48.7	
Richest	48.7	
**Religion**		< 0.001
Hindu	59.6	
Muslims	67.1	
Sikh	56.1	
Others	64.0	
**Caste**		< 0.001
Scheduled Caste	66.2	
Scheduled Tribe	71.4	
Other Backward Class	56.5	
Others	58.3	
**Place of residence**		< 0.001
Rural	63.0	
Urban	51.6	
**State**		< 0.001
Himachal Pradesh	54.2	
Punjab	54.9	
West Bengal	81.9	
Orissa	69.3	
Maharashtra	55.0	
Kerala	66.3	
Tamil Nadu	40.7	
**Total**	60.0	

*p*-value based on chi-square test; *IADL* Instrumental activities of daily living, *ADL* Activities of daily living

**Fig. 2:**
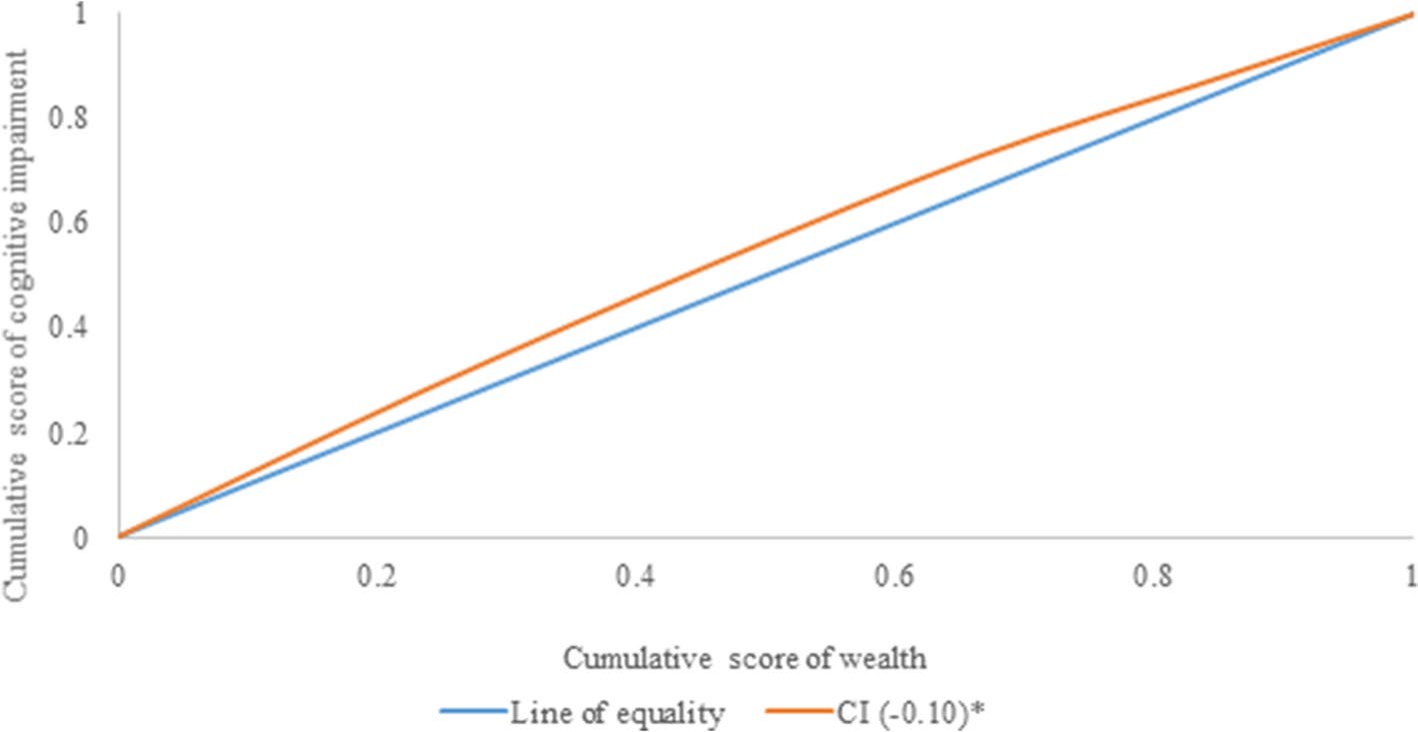
Concentration curve for cognitive impairment among older adults. *CCI: Concentration Index; *If *p* < 0.05

Figure 2 reveals the concentration curve for cognitive impairment among older adults. It was found that cognitive impairment was concentrated among older adults from households with the lowest wealth quintile (CCI: − 0.10: *p* < 0.05).

Table 3 presents estimates of decomposition analysis for the contribution of various explanatory variables for cognitive impairment among older adults.

**Table 3:** Estimates of decomposition analysis for contribution of various explanatory variables for cognitive impairment among older adults

Variables	Coef. (95% CI)	Elasticity	CCI	Absolute contribution to CCI	% contribution	
**Self-perceived income sufficiency**						
Has income and fully sufficient	Ref.					
Has income and partially sufficient	0.33*(0.19,0.46)	0.018	−0.065	− 0.001	2.5	3.0
Has income and not sufficient	0.29*(0.07,0.52)	0.003	− 0.121	0.000	0.8	
No income	0.22*(0.07,0.37)	0.013	0.014	0.000	−0.4	
**Work Status (last one year)**						
Never worked	Ref.					
Currently working	−0.13(− 0.28,0.02)	− 0.010	− 0.176	0.002	−3.9	0.7
Retired	− 0.26*(− 0.46,-0.07)	− 0.004	0.516	−0.002	4.6	
**Educational status**						
Not educated	Ref.					
Below 5 years	−0.37*(− 0.51,-0.24)	−0.011	− 0.001	0.000	0.0	44.6
6 to 10 years	−0.80*(− 0.94,-0.66)	−0.041	0.259	−0.011	23.0	
11+ years	−1.27*(−1.50,-1.04)	−0.016	0.621	−0.010	21.5	
**Marital status**						
Not in union	Ref.					
Currently in union	−0.17*(−0.28,-0.05)	− 0.019	0.039	− 0.001	1.6	1.6
**Asset ownership**						
No	Ref.					
Yes	−0.19*(− 0.33,-0.06)	−0.030	0.025	−0.001	1.6	1.6
**Age group (in years)**						
60-69	Ref.					
70-79	0.39*(0.28,0.5)	0.019	−0.014	0.000	0.6	0.0
80+	0.65*(0.46,0.84)	0.012	0.024	0.000	−0.6	
**Gender**						
Men	Ref.					
Women	0.04(−0.09,0.16)	0.009	−0.033	0.000	0.6	0.6
**Co-residing with children**						
No	Ref.					
Yes	−0.05(−0.16,0.06)	−0.008	0.090	−0.001	1.5	1.5
**Self-rated health**						
Good	Ref.					
Poor	0.25*(0.14,0.35)	0.033	−0.038	−0.001	2.7	2.7
**IADL**						
High	Ref.					
Low	0.23*(0.12,0.33)	0.028	−0.062	−0.002	3.7	3.7
**ADL**						
High	Ref.					
Low	0.65*(0.42,0.88)	0.005	−0.011	0.000	0.1	0.1
**Subjective well-being**						
High	Ref.					
Low	0.12*(0.02,0.25)	0.004	−0.283	−0.001	2.5	2.5
**Psychological health**						
High	Ref.					
Low	0.46*(0.32,0.6)	0.023	−0.233	−0.005	11.5	11.5
**Wealth status**						
Poorest	Ref.					
Poorer	0.08(−0.08,0.24)	0.002	−0.338	−0.001	1.7	31.8
Middle	−0.07(− 0.24,0.11)	−0.001	0.140	0.000	0.4	
Richer	−0.34*(− 0.53,-0.15)	−0.014	0.522	−0.007	16.2	
Richest	−0.36*(− 0.57,-0.16)	−0.008	0.763	−0.006	13.6	
**Religion**						
Hindu	Ref.					
Muslims	−0.1(−0.31,0.11)	−0.002	0.148	0.000	0.7	−1.5
Sikh	−0.08(− 0.31,0.16)	0.000	0.296	0.000	−0.3	
Others	0.26*(0.02,0.5)	0.003	0.319	0.001	−1.9	
**Caste**						
Scheduled Caste	Ref.					
Scheduled Tribe	0.01(−0.24,0.26)	0.002	−0.445	− 0.001	2.2	3.7
Other Backward Class	−0.02(− 0.17,0.13)	0.005	− 0.029	0.000	0.3	
Others	−0.04(− 0.18,0.11)	−0.002	0.222	−0.001	1.2	
**Place of residence**						
Rural	Ref.					
Urban	−0.10(−0.21,0.01)	0.000	0.253	0.000	0.1	
**State**						
Himachal Pradesh	Ref.					
Punjab	0.14(−0.07,0.36)	0.000	0.318	0.000	0.2	−8.2
West Bengal	1.48*(1.27,1.7)	0.028	−0.162	−0.005	10.0	
Orissa	0.50*(0.32,0.69)	0.011	−0.366	−0.004	8.6	
Maharashtra	0.14(−0.04,0.32)	−0.001	− 0.116	0.000	− 0.2	
Kerala	0.85*(0.66,1.05)	0.022	0.351	0.008	−16.4	
Tamil Nadu	−0.53*(−0.74,-0.33)	− 0.022	−0.217	0.005	−10.4	
**Calculated CCI**				−0.046	100.0	100.0
**Actual CCI**				−0.100		
**Residual**				−0.054		

*CI* Confidence interval, *CCI* Concentration index, *if *p* > 0.05, % Percentage, *IADL* Instrumental activities of daily living, *ADL* Activities of daily living

The coefficients in the table are the regression coefficients with 95% confidence interval (CI) to represent how cognitive impairment is associated with other explanatory variables. For instance, it was found that the likelihood of cognitive impairment was high among older adults with a low level of self-perceived income sufficiency [coefficient: 0.29; CI: 0.07- 0.52] compared to older adults with higher levels of perceived income status. On the other hand, older adults who were retired had a lower likelihood of cognitive impairment [coefficient: -0.26; CI: − 0.46- -0.07] in comparison to older adults who never worked in the last year. Similarly, older adults with more than 10 years of education were less likely to be cognitively impaired [coefficient: -1.27; CI: − 1.50- -1.04] in comparison to those with no education.

The CCI indicates concentration index, and negative CCI denotes that cognitive impairment was concentrated among economically poor older adults for that particular predictor and vice-versa. The absolute contribution is the product of elasticity and CCI whereas the percentage contribution is the proportion of absolute contribution multiplied by 100. Educational status, wealth status, and psychological health were the significant factors that contributed to the inequality for cognitive impairment among older adults =. For instance, educational status among older adults explained 44.6% of socioeconomic inequality, followed by 31.8% by wealth status and 11.5% by psychological health. Apart from these factors, IADL (3.7%), caste (3.7%), and self-perceived income sufficiency (3.0%) explained socioeconomicinequality in cognitive impairment among older adults.

## Discussion

In the present study, we found a higher concentration of cognitive impairment among older Indian adults from poor socioeconomic backgrounds. As evidence suggests, self-perceived income sufficiency is considered a useful tool to assess resources and health disparities in underserved populations [14, 42]. The results of the present study also show that subjective income status measured by self-perceived income sufficiency had a significant association in cognitive functioning in older ages. It is noteworthy that the measure of subjective income status has been used in past studies to assess the economic well-being as well as satisfaction and stress levels [42, 43], and is recommended to assess the resource availability among underserved populations [14]. In a study using data from World Health Organization’s Study on global AGEing and adult health (SAGE), perceived income adequacy was found to be significantly associated with self-rated health among older adults [44]. Again, our results are consistent with studies in less-developed societies that found a significant positive association of late-life perceived insufficient income with cognitive impairment [45].

Further, older Indian adults often tend to work beyond the age of retirement due to a lack of pension and social security in the informal labour market [46, 47]. However, although the result was not significant, the current analysis showed a possible beneficial effect of working status in the last year on cognitive abilities. However, cognitive difficulties were found to reduce individuals’ ability to work in multiple studies [48, 49], and the current finding may be explained by the possible reverse causality in the association, where older adults who are cognitively healthy may continue to work in later life [25]. Also, as evidence suggests, the protective effects of the reserve may be established early in life, before people enter the workforce [50], suggesting that the work status of the older individual may not affect his/her cognitive health but the age maybe a common cause in such association. As documented, after retirement, older adults may enjoy better health conditions and have more time to spend in social life [51]. Consistently, the present study found a significant negative association of retirement with cognitive impairment. The finding is substantiated by the theory of “relieved effect on mental functioning”, which suggests that retirement from a stressful occupation may reduce mental worries [25]. While examining the direction of the effect between retirement and cognitive functioning, multiple studies have revealed that poor physical, mental and cognitive health may affect retirement decisions and lead to the early retirement of older individuals [52–54].

Our finding that higher levels of education act as a major contributing factor to higher cognitive performance is consistent with previous studies [7, 18, 55, 56]. Further, the substantial contribution of lower levels of education to the cognitive health disparities can be explained as increased resource availability and access to higher education may mediate one’s health behaviours to some extent and could enhance his/her overall health, especially cognitive functioning [57]. It is again explained by the hypothesis of ‘brain reserve capacity that argues that those people with higher levels of education may have a larger brain reserve capacity than people with no or low levels of education [58]. It is also shown that more educated people tend to experience less cognitive decline because high educational attainment is a protective factor against neuropathology [11]. In the decomposition analysis, we found the largest contribution of education to cognitive health inequalities, with higher education contributing more to increased socioeconomic inequality in cognitive impairment. Another study found education as contributing to the capacity for cerebral reserves [59]. Furthermore, in addition to biological effects, education can also increase competencies and enhance cognitive abilities [17]. These can all lead to improved cognitive abilities and cognitive networking efficiency among older adults.

Although education overwhelmingly determines the pathway, studies show that household wealth has the same causal linkage as education, by which cognitive ability could be enhanced [19]. The study also found a significant contribution of household wealth index and asset ownership to socioeconomic inequalities in cognition. Previous studies support the association of household economic status with older individuals’ cognitive abilities and the contribution of household factors to the cognitive inequalities among the ageing population [21, 24, 60, 61]. Such an association of higher economic status that associates a greater cognitive ability could partially be explained by the fact that having material possessions or equipment appears to be beneficial for cognitive functioning in terms of reduced risk of indoor air pollution. The poor economic status triggering cognitive deficits may also be attributed to the earlier evidence that shows that lack of material resources may result in increased stress or inflammation and poor neural health that leads to cognitive deficits [62].

Evidence from studies of cognitive function and marital status indicates that, for both genders, married people are less likely to suffer from dementia than those who are divorced, separated, or single [63]. Decomposing the factors in the present study has shown that marital status had a significant contribution to cognitive inequalities among older adults. Also, married individuals are at lower risk of developing cognitive problems mainly because of better mental conditions and lifestyle behaviours [64, 65].

Furthermore, all the health-related variables in the current study show significant contributions to the inequalities in old age cognitive impairment. Among all, the substantial contribution of poor psychological health shows a lack of a healthy brain that can stimulate the cerebral nervous system and positively affect cognitive health, which may worsen SES-related inequality in cognitive functioning among an aging population. Consistent with earlier studies [60, 66], difficulty with two or more activities of daily living among study participants was also shown as a significant factor that intensifies the inequalities in cognitive functioning. However, functional ability in many studies also has been documented to be associated with cognition with reverse causation, where older adults with higher cognitive abilities may have better functional health [67, 68]. Finally, the higher prevalence of cognitive impairment in the states of West Bengal, Odisha and Kerala suggest the need for future studies focusing on regional variations in late-life cognitive impairment in India.

The study has the merit of decomposing the contribution of several socioeconomic and health-related factors in socioeconomic inequalities in late-life cognitive health using a large survey data. However, the study has certain limitations too. Firstly, cognitive impairment is measured only through the word recall method and did not consider other measures of cognitive functioning such as orientation and executive and arithmetic functioning. Although the analysis was adjusted for education, assessing the cognitive abilities using word recall method in a population with higher rate of illiteracy (50.7% older adults with no formal education in the current study) might have resulted in higher prevalence of cognitive impairment and may bias the current findings. Secondly, the estimates provided are just the association of the past as the survey was conducted in 2011, and further investigation is required using more recent datasets. Lastly, the respondents were selected from seven states of India, which represent different regions of India; therefore, one should be cautious while generalizing it for pan India. Apart from limitations, the survey was well structured and focused entirely on the issues of older adults; hence the estimates and associations are reliable.

## Conclusions

Findings suggest that older adults with lower perceived income, lower levels of education, poor physical and mental health, and poor physical and social resources were more likely to be cognitively impaired. Hence, these factors can be adopted further to evaluate the health inequalities and develop better policies and programs for a dementing population. Education, wealth and psychological health are major contributors in socioeconomic inequality in late-life cognitive impairment in the current study, which may be target areas in future policy formulation to reduce the inequality in cognitive impairment in older Indian adults.

The finding that education has a significant largest contribution to cognitive impairment late in life which is in line with existing studies, suggests that educational attainment may bring positive changes in the fundamental brain functions and allow older adults to cope up with the age-related cognitive changes [69, 70]. This has particular significance in the Indian context, where a major chunk of older adults are illiterate. Further longitudinal studies are warranted to investigate the factors contributing to differential declines in cognition and establish causal relationships between associated factors and cognitive impairment.

## Data Availability

The data that support the findings of this study are available from [director@isec.ac.in or india.office@unfpa.org] but restrictions apply to the availability of these data, which were used under license for the current study, and so are not publicly available. Data are however available from the authors upon reasonable request and with permission of [director@isec.ac.in or india.office@unfpa.org].

## Abbreviations

ADL: Activities of Daily Living
IADL: Instrumental Activities of Daily Living
SC: Scheduled Caste
ST: Scheduled Tribe
CI: Confidence interval
CCI: Concentration index
